# Physical activity and risk of cardiovascular disease by weight status among U.S adults

**DOI:** 10.1371/journal.pone.0232893

**Published:** 2020-05-08

**Authors:** Xiaochen Zhang, Rebecca E. Cash, Julie K. Bower, Brian C. Focht, Electra D. Paskett

**Affiliations:** 1 Division of Population Sciences, The Ohio State University Comprehensive Cancer Center, Columbus, OH, United States of America; 2 Division of Epidemiology, The Ohio State University College of Public Health, Columbus, OH, United States of America; 3 Department of Emergency Medicine, Massachusetts General Hospital, Boston, MA, United States of America; 4 Division of Kinesiology, Department of Human Sciences, The Ohio State University, Columbus, OH, United States of America; Pennington Biomedical Research Center, UNITED STATES

## Abstract

**Purpose:**

We sought to determine whether the association between physical activity and 10-year cardiovascular disease (CVD) risk varies among normal weight, overweight, and obese adults in a nationally-representative sample of the United States.

**Methods:**

Data were from the National Health and Nutrition Examination Survey 2007–2016. A subset of 22,476 participants aged 30–64 years was included with no CVD history. Physical activity level was self-reported and stratified into sedentary (0 min/week), inactive (1–149 mins/week), or active (≥150 mins/week) of moderate or vigorous activities. Framingham risk scores were classified as low/intermediate (<20%) or high 10-year CVD risk (≥20%).

**Results:**

The average age of the population was 45.9 years, 52.3% were female, 33.6% were overweight (BMI 25.0–29.9kg/m^2^), and 35.7% were obese (BMI≥30kg/m^2^). Individuals who were overweight and obese had a higher 10-year CVD risk compared to those with normal weight (9.5 vs. 10.1 vs. 6.3%, P<0.001). The association of physical activity and high 10-year CVD risk differed by weight status. Among overweight and obese adults, individuals engaged in any physical activity had lower odds ofhigh 10-year CVD risk compared to sedentary individuals (overweight: OR active = 0.48, 95% CI: 0.36–0.64; OR inactive = 0.53, 95% CI: 0.45–0.86; obese: OR active = 0.50, 95% CI: 0.37–0.68; OR inactive = 0.66, 95% CI: 0.49–0.89). Among normal weight adults, individuals who were physically active had lower odds of high 10-year CVD risk (OR = 0.59, 95% CI: 0.28–0.87). When compared the joint effects of physical activity level and weight status, physical activity was associated with a larger magnitude of reduced odds of 10-year CVD risk than weight status.

**Conclusion:**

Participation in any level of physical activity is associated with a lower 10-year CVD risk for overweight and obese adults. Future studies are needed to identify effective modes and doses of exercise that offer optimal CVD benefits for populations with different weight statuses.

## Introduction

In 2015–2016, 39.8% of U.S adults were obese and 31.8% were overweight [1]. Obesity is soon replacing smoking as the leading cause of preventable premature death in the U.S [2], as it is a major risk factor for cardiovascular disease (CVD), type 2 diabetes, hypertension, and cancer [3–5]. Excessive adiposity accumulates and alters cardiac structure and function in addition to metabolic dysfunction, even in the absence of comorbidities [6]. Thus, overweight and obesity may affect the heart through the effect on known risk factors such as dyslipidemia, hypertension, glucose intolerance, and inflammatory markers, as well as other unrecognized mechanisms [5].

A sedentary lifestyle is a major modifiable risk factor for CVD [3, 7–10]. Many organizations, including the American Heart Association and the American College of Sports Medicine, have recommended increasing physical activity or aerobic exercise training to increase levels of cardiorespiratory fitness in the general population [3, 7, 11]. The updated Physical Activity Guidelines for Americans emphasize that moving more and sitting less will benefit nearly everyone [7]. Especially for sedentary individuals, even increasing a small amount of physical activity can provide CVD benefits, lower the risk of coronary heart disease, and reduce all-cause and CVD-specific mortality [9, 10, 12]. Higher levels of physical activity can attenuate elevated cardiovascular morbidity and mortality in obese adults with and without underlying CVD [9, 10, 13]. Intensive Lifestyle Interventions that combine increased physical activity and calorie-restricted diet have been proposed in overweight and obese populations and demonstrate favorable effects on cardiovascular risk factors, such as decreased insulin resistance, blood pressure, and inflammatory markers, and improved lipid profiles [14]. Although epidemiological studies report conflicting results of weight loss and risk reduction of CVD morbidity and mortality among obese adults [14–16], individuals with favorable behavioral responses might be more likely to benefit from ILIs and have a lower long-term CVD risk [17].

Being at-risk of CVD, individuals with overweight and obesity can increase physical activity to avoid a sedentary lifestyle, with a goal of obtaining a healthy weight and reducing CVD risk. However, obesity is associated with variations of physiological functions and metabolic characteristics, which may influence the response to physical activity [18–21]. It is unknown whether overweight and obese adults receive the same health benefits from physical activity compared to adults with normal weight. It is possible that individuals who are obese may respond differently to the same level of physical activity compared to individuals with normal weight, due to differences in body composition and energy expenditure [18, 19, 21]. Thus, we aimed to determine 1) the 10-year CVD risk across U.S adults with normal weight, overweight, and obesity; and 2) whether the association of physical activity and CVD risk varies across weight statuses in a nationally-representative sample.

## Materials and methods

We used data from the National Health and Nutrition Examination Survey (NHANES) 2007–2016. NHANES is a cross-sectional survey that uses a stratified, multistage probability sampling approach designed to represent the non-institutionalized U.S population, with oversampling of minority groups. All participants provided written informed consent [22]. The current study included adults aged 30–64 years old without a history of CVD, with completed data on height, weight, and CVD risk factors. The presence of CVD was self-reported if a doctor had ever told participants that they had any of the following: myocardial infarction, congestive heart failure, stroke, and coronary disease. BMI was calculated by study technicians using standard measures of height (meters) and weight (kg). Participants with a BMI <25.0 kg/m^2^, between 25.0–29.9 kg/m^2^, and BMI ≥30 kg/m^2^ were classified as normal weight/underweight, overweight, and obese, respectively.

Daily activities and leisure time activities were measured based on the Global Physical Activity Questionnaire [23]. Physical activity was quantified using the self-reported frequency of vigorous and moderate recreation activities (at least 10 minutes continuously) in a typical week. According to published guidelines, participants who reported 0, 1–149, or ≥150 minutes per week of physical activity were classified as sedentary, physically inactive, and physically active, respectively [3, 7, 11, 24]

We implemented the Framingham Risk Scores (FRS) to estimate 10-year CVD risk [25]. The total score was calculated based on participants’ age, high-density lipoprotein cholesterol (HDL-C), total cholesterol (TC), systolic blood pressure, smoking status, and diabetes, stratified by gender. HDL-C, TC, and systolic blood pressure were measured by study technicians during the physical examination. Diabetes was determined based on self-reported medical conditions or medication use for diabetes. Smoking status was defined as a current smoker or not current smoker (including both former and never smokers). The 10-year CVD risk was then determined using the total score. Participants with an FRS ≥20% were considered to have high CVD risk, and FRS <20% was considered as low/intermediate CVD risk.

Demographic characteristics including age, sex, race, marital status, education, and income to poverty ratio were self-reported using a standardized questionnaire. A dietary interview was conducted to measure detailed dietary intake information for each participant. Dietary intake was assessed according to the Life Simple’s 7 Healthy Diet metric [24, 26]. Specifically, dietary intake in five components was evaluated: ≥4.5 cups fruit/vegetable per day; ≥ three 1-oz whole grain per day; <1,500 mg of sodium per day; ≥ two 3.5-oz servings of fish per week; and <450 calories from sugared drinks per week. Participants with 0–1 components or 2–5 components were classified as poor diet or intermediate/ideal diet, respectively, due to limited participants meeting ideal diet criteria. A depression score was calculated using the nine-item Patient Health Questionnaire (PHQ-9) to determine the frequency of depressive symptoms over the past two weeks [27].

All analyses incorporated the NHANES sample weights and accounted for the complex sample survey design using standard methods [28]. Continuous variables were presented as weighted means ± SE, and categorical variables were presented as weighted frequency (%). We used ANOVA and Chi-square tests to compare continuous and categorical variables across weight status (normal weight/underweight, overweight, and obese). Unconditional logistic regression quantified the association of physical activity and high CVD-risk and stratified by weight status. Models were estimated as unadjusted (model 1), adjusted for demographic characteristics including race, marital status, education level, and income-to-poverty ratio (model 2), and fully adjusted for demographic, dietary intake, and depressive symptoms score (model 3). The interaction between physical activity level and weight status was included in each model and tested using the adjusted Wald test. We examined the association of physical activity level and 10-year CVD risk within each weight status and also compared the joint effect of each combination of physical activity level and weight status among sedentary obese adults on 10-year CVD risk. The odds ratios (OR) and 95% confidence interval (95% CI) were estimated from separate models, stratified by weight status. Statistical significance for interaction was evaluated as P<0.10 and for all other analyses was P<0.05 [29]. All statistical analyses were completed using Stata MP Version 15.1 (StataCorp, College Station, TX) in July 2019.

## Results

We identified 24,913 adults aged 30 to 64 years with no CVD history. Sufficient information was available on 22,476 participants to define the CVD risk according to the Framingham Risk Score. Participants with insufficient information necessary to define CVD risk were more likely to be Non-Hispanic Black (18.3% vs. 10.6%, P<0.001), high school or less (44.6% vs. 37.4%, P<0.001), never married (25.1% vs. 18.8%, P<0.001), and less than 138% income to poverty ratio (31.8% vs. 23.1%, P<0.001), compared to those with sufficient information necessary to define CVD risk, respectively (data not shown).

In our study population, after applied sample weights, the average age was 45.9 years old, 30.7% were normal weight/ underweight, 33.6% were overweight, and 35.7% were obese. The majority were non-Hispanic whites (67.0%), and 52.3% were female **(Table 1)**. Compared to participants with BMI <25 kg/m^2^, participants who were overweight were more likely to be male (55.0% vs. 41.8%), more likely to be married (67.9% vs. 60.0%), less likely to be a college graduate or above (32.1% vs. 37.2%), have a higher depression score (1.46 ± 0.02 vs. 1.43 ± 0.02), and more likely to have a poor diet (72.3% vs. 70.5%), and to be sedentary (42.5% vs. 37.2%). The characteristics of participants who were obese were in the same direction with those who were overweight, but they were more likely to be Non-Hispanic Black (14.0% vs. 8.6%) and less likely to have >400% income-to-poverty ratio (33.5% vs. 38.8%), compared to participants with BMI <25 kg/m^2^.

**Table 1:** Characteristics of U.S adults age 30–65 years old with no history of cardiovascular disease (population-weighted, NHANES 2007–2016).

	Total	Normal weight	Overweight	Obese	P value
	n = 22,476^a^	n = 6,683 (30.7%) ^a^	n = 7,477 (33.6%) ^a^	n = 8,316 (35.7%) ^a^	
Age, years	45.88±0.25	43.22±0.40	47.36±0.29	46.75±0.27	<0.001
Sex, %					<0.001
Male	47.7%	41.8%	55.0%	45.8%	
Female	52.3%	58.2%	45.0%	54.2%	
Race, %					<0.001
Non-Hispanic White	67.0%	68.7%	68.3%	64.3%	
Non-Hispanic Black	10.6%	8.6%	8.9%	14.0%	
Other ^b^	22.4%	22.8%	22.8%	21.6%	
Marital Status, %					<0.001
Married	64.2%	60.0%	67.9%	64.3%	
Widowed/divorced	17.0%	15.3%	16.6%	18.8%	
Never married	18.8%	24.6%	15.5%	16.9%	
Education, %					<0.001
High school or less	37.4%	33.2%	38.1%	40.3%	
Some college	31.9%	29.7%	29.8%	35.8%	
College graduate or more	30.7%	37.2%	32.1%	23.9%	
Income to Poverty Ratio, %					<0.001
<138%	23.1%	23.2%	21.7%	24.4%	
138–250%	19.6%	18.5%	18.6%	21.4%	
250–400%	19.8%	19.5%	19.3%	20.7%	
>400%	37.4%	38.8%	40.4%	33.5%	
Depression Score	1.49±0.01	1.43±0.02	1.46±0.02	1.56±0.02	<0.001
Poor Diet, %					
No	27.0%	30.5%	27.7%	23.4%	
Yes	73.0%	70.5%	72.3%	76.6%	
Physical Activity Level, %						<0.001
Sedentary	44.4%	37.2%	42.5%	52.5%	
Inactive	16.5%	16.3%	16.4%	16.9%	
Active	39.0%	46.5%	41.2%	30.6%	

a. The sample size (n) was unweighted; percentages were weighted

b. other race includes: Mexican American, Other Hispanic, Non-Hispanic Asian, and multi-racial

In our study population, 12.3% of participants were classified as high 10-year CVD risk. Specifically, 8.0% of individuals who were BMI <25 kg/m^2^, 13.6% of individuals who were overweight, and 14.7% of individuals who were obese were classified with high 10-year CVD risk (P<0.001, **Table 2**). Similarly, the mean estimated 10-year CVD risk score (FRS) was lowest among individuals with BMI <25 kg/m^2^ (6.30 ± 0.18), followed by individuals who were overweight (9.53 ± 0.19), and highest among individuals who were obese (10.13 ± 0.17). In terms of individual CVD risk factors from the FRS criteria, compared to those with BMI <25 kg/m^2^, participants who were overweight or obese were more likely to report poorer levels of HDL-C, total cholesterol, systolic blood pressure, as well as being a smoker and having diabetes (all P<0.001). However, no significant difference was observed in age distribution across weight status (P *interaction* = 0.107).

**Table 2:** Estimated cardiovascular Risk and individual risk factors among U.S adults age 30–65 years old with no history of cardiovascular disease (population-weighted, NHANES 2007–2016).

	Total	Normal weight	Overweight	Obese	P value
***Estimated CVD Risk***					<0.001
10-yr CVD risk, points	8.75±0.15	6.30±0.18	9.53±0.19	10.13±0.17	
High-risk CVD	12.3%	8.0%	13.6%	14.7%	<0.001
***Individual Risk Factors***					
Age Group, years					0.107
30–34	14.4%	16.1%	14.2%	13.4%	
35–39	14.6%	15.5%	13.7%	14.7%	
40–44	15.0%	15.0%	15.0%	15.0%	
45–49	15.8%	16.2%	16.3%	15.1%	
50–54	15.4%	14.5%	15.8%	15.7%	
55–59	13.6%	13.3%	13.6%	13.8%	
60–64	11.2%	9.5%	11.5%	12.3%	
HDL-C (mmol/L)					<0.001
>1.6	25.4%	42.4%	22.7%	13.3%	
1.3–1.6	26.5%	29.3%	28.0%	22.6%	
1.2–1.29	10.9%	8.7%	11.7%	11.9%	
0.9–1.19	28.3%	16.7%	28.9%	37.9%	
<0.9	8.9%	2.9%	8.7%	14.4%	
Total Cholesterol(mmol/L)					<0.001
<4.1	17.9%	21.8%	15.2%	17.0%	
4.1–5.19	40.4%	42.8%	38.4%	40.2%	
5.2–6.19	28.5%	25.1%	30.3%	29.6%	
6.2–7.2	10.5%	8.3%	12.2%	10.6%	
>7.2	2.9%	1.9%	3.9%	2.6%	
Systolic Blood Pressure (mmHg)					<0.001
<120	52.1%	64.3%	51.7%	42.0%	
120–129	22.6%	17.2%	23.6%	26.4%	
130–139	12.5%	8.7%	12.4%	15.8%	
140–149	6.6%	4.6%	6.8%	8.2%	
150–159	3.0%	2.1%	2.8%	3.9%	
≥160	3.2%	3.0%	2.8%	3.7%	
Smoker					<0.001
No	80.0%	76.2%	81.3%	82.4%	
Yes	20.0%	23.8%	18.7%	17.6%	
Diabetes					<0.001
No	91.9%	96.9%	93.6%	85.9%	
Yes	8.1%	3.1%	6.4%	14.1%	

CVD: cardiovascular disease

Sample weights were applied

The association of physical activity level and high-risk of CVD differed by weight status, engaging in any physical activity was associated with lower odds of high 10-year CVD risk among overweight and obese adults (P-interaction for all 3 models <0.1, **Table 3**). In the unadjusted model (**model 1**), compared to sedentary adults, adults who were inactive or active had lower odds of high 10-year CVD risk, regardless of weight status. The magnitudes of the associations were greater among those who were active compared to those who were inactive. Similarly, when adjusted for race/ethnicity, marital status, education level, and household income to poverty ratio (**model 2**), adults who were inactive or active had lower odds of high 10-year CVD risk compared to those who were sedentary, regardless of weight status. After further adjustment for the dietary intake and depression score (**model 3**), among adults with BMI <25 kg/m^2^ (compared to those who were sedentary), adults who were inactive were 60% lower odds of high 10-year CVD risk (OR = 0.40, 95% CI: 0.29–0.56), but no difference was observed among those who were inactive (OR = 0.74, 95% CI: 0.48–1.13). Among adults who were overweight (compared to those who were sedentary), adults who were inactive and active had 47% and 52% lower odds of high 10-year CVD risk (OR = 0.53, 95% CI: 0.45–0.86; OR = 0.48, 95% CI: 0.36–0.64, respectively). Similarly, among adults who were obese (compared to those who were sedentary), adults who were inactive and active had 34% and 50% lower odds of high 10-year CVD risk (OR = 0.66, 95% CI: 0.49–0.89; OR = 0.50, 95% CI: 0.37–0.68, respectively).

**Table 3:** The association of physical activity level and high 10-year CVD risk by weight status among U.S adults age 30–65 years old with no history of cardiovascular disease (population-weighted, NHANES 2007–2016).

	Normal weight			Overweight			Obese		
	OR	95% CI	P value	OR	95% CI	P value	OR	95% CI	P value
**Model 1**									
Physical Activity Level									
Sedentary	reference	--	--	reference	--	--	reference	--	--
Inactive	0.55	0.41, 0.74	<0.001	0.68	0.52, 0.88	0.004	0.61	0.49, 0.77	<0.001
Active	0.32	0.26, 0.40	<0.001	0.45	0.37, 0.54	<0.001	0.49	0.40, 0.60	<0.001
***P interaction<0*.*001***									
**Model 2**									
Physical Activity Level									
Sedentary	reference	--	--	reference	--	--	reference	--	--
Inactive	0.68	0.50, 0.82	0.013	0.71	0.55, 0.93	0.013	0.60	0.47, 0.76	<0.001
Active	0.46	0.35, 0.59	<0.001	0.53	0.43, 0.66	<0.001	0.53	0.43, 0.65	<0.001
***P interaction<0*.*001***										
**Model 3**									
Physical Activity Level									
Sedentary	reference	--	--	reference	--	--	reference	--	--
Inactive	0.74	0.48, 1.13	0.16	0.53	0.45, 0.86	0.01	0.66	0.49, 0.89	0.01
Active	0.40	0.29, 0.56	<0.001	0.48	0.36, 0.64	<0.001	0.50	0.37, 0.68	<0.0001
***P interaction<0*.*001***									

**Model 1:** unadjusted; **Model 2:** adjusted for race/ethnicity, marital status, education level, and income to poverty ratio; **Model 3:** adjusted for race/ ethnicity, marital status, education level, income to poverty ratio, diet intake, and depression score. **OR:** odds ratio; **95% CI:** 95% confidence interval. Interaction between physical activity level and weight status was included in each model and tested using the adjusted Wald test. OR and 95% CI were calculated in separate models, stratified by weight status. Sample weights were applied to all models.

Additional analyses that compared the joint effects of physical activity level and weight status on 10-year CVD risk showed that compared to sedentary obese U.S adults, inactive obese and active obese adults had 34% (OR _obese &inactive_ = 0.66, 95%CI: 0.49, 0.89) and 50% (OR _obese &active_ = 0.50, 95%CI: 0.37, 0.69) lower odds of high 10-year CVD risk, respectively **(Table 4, model 3)**. Similarly, within overweight and normal weight categories, physically active and inactive adults had lower odds of high 10-year CVD risk compared to sedentary adults. The magnitude of reduced odds were larger among physically active adults (OR _normal weight &active_ = 0.22, OR _overweight &active_ = 0.47), followed by physically inactive adults (OR _normal weight &inactive_ = 0.42, OR _overweight &inactive_ = 0.62). Whereas compared to sedentary obese U.S adults, sedentary normal weight adults had 40% (OR _normal weight &sedentary_ = 0.60, 95%CI: 0.47, 0.77) lower odds of high-10-year CVD risk. On the other hand, within inactive and active adults, the magnitude of reduced odds of high 10-year CVD risk were larger among normal weight adults (OR _normal weight &active_ = 0.22, OR _normal weight &inactive_ = 0.42) compared to obese adults (OR _obese &active_ = 0.50, OR _obese &inactive_ = 0.66). However, the odds ratio for high 10-year CVD risk were similar between overweight and obese adults within each physical activity level.

**Table 4:** Comparison of joint effects of physical activity level and weight status on high 10-year CVD risk among U.S adults age 30–65 years old with no history of cardiovascular disease (population-weighted, NHANES 2007–2016).

Physical Activity Level	Weight Status	OR	95% CI	P value
**Model 1**				
Sedentary	Obese	Reference	--	--
Sedentary	Overweight	0.99	0.87, 1.13	0.935
Sedentary	Normal weight	0.65	0.55, 0.77	<0.001
Inactive	Obese	0.61	0.49, 0.77	<0.001
Inactive	Overweight	0.68	0.52, 0.88	0.004
Inactive	Normal weight	0.36	0.26, 0.49	<0.001
Active	Obese	0.49	0.40, 0.60	<0.001
Active	Overweight	0.45	0.38, 0.52	<0.001
Active	Normal weight	0.21	0.17, 0.26	<0.001
***P***_***interaction***_***<0*.*001***				
**Model 2**				
Sedentary	Obese	Reference	--	--
Sedentary	Overweight	0.99	0.88, 1.14	0.915
Sedentary	Normal weight	0.67	0.55, 0.81	<0.001
Inactive	Obese	0.62	0.49, 0.78	<0.001
Inactive	Overweight	0.70	0.54, 0.91	0.007
Inactive	Normal weight	0.42	0.31, 0.58	<0.001
Active	Obese	0.55	0.45, 0.68	<0.001
Active	Overweight	0.52	0.44, 0.62	<0.001
Active	Normal weight	0.27	0.21, 0.34	<0.001
***P***_***interaction***_***<0*.*001***				
**Model 3**				
Sedentary	Obese	Reference	--	--
Sedentary	Overweight	0.99	0.81, 1.21	0.947
Sedentary	Normal weight	0.60	0.47, 0.77	<0.001
Inactive	Obese	0.66	0.49, 0.89	<0.001
Inactive	Overweight	0.62	0.44, 0.86	0.005
Inactive	Normal weight	0.42	0.28, 0.64	<0.001
Active	Obese	0.50	0.37, 0.69	<0.001
Active	Overweight	0.47	0.36, 0.61	<0.001
Active	Normal weight	0.22	0.16, 0.29	<0.001
***P***_***interaction***_***<0*.*001***				

**Model 1:** unadjusted; **Model 2:** adjusted for race/ethnicity, marital status, education level, and income to poverty ratio; **Model 3:** adjusted for race/ ethnicity, marital status, education level, income to poverty ratio, diet intake, and depression score. **OR:** odds ratio; **95% CI:** 95% confidence interval. Interaction between physical activity level and weight status was included in each model and tested using the adjusted Wald test. Sample weights were applied to all models.

## Discussion

In our analyses of adults aged 30–64 years with no CVD history, adults who were overweight or obese were more likely to have a high 10-year CVD risk compared to adults with BMI <25 kg/m^2^. Independent of race/ethnicity, social-economic status, dietary intake, and depression, engaging in any physical activity was associated with a lower odds of high 10-year CVD risk among adults who were overweight or obese. Whereas, among adults with BMI <25 kg/m^2^, being physically active (meeting physical activity guidelines) was associated with a lower odds of high 10-year CVD risk. Our finding is consistent with previous studies in other populations that overweight/obesity is associated with higher CVD risk and increasing even a small amount of physical activity is linked with a lower risk of CVD risk within each weight status [9, 10, 30–32]. Moreover, we further examined the interaction between physical activity and weight status on CVD-risk, which confirmed our hypothesis that cardiovascular benefits from physical activity vary among adults with normal weight, overweight, and obesity. Additionally, when we compared the joint effects of physical activity level and weight status, we found that physical activity was associated with a larger magnitude of reduced odds of 10-year CVD risk than weight status. This is consistent with recent evidence that physical activity plays a more important role than weight status or weight loss in CVD-specific and all-cause mortality [32, 33]. Therefore, increasing physical activity level, especially promoting to meet the physical activity guidelines, could provide cardiovascular benefits for adults regardless of weight status [9, 10].

Intensive lifestyle interventions (ILI) combining a calorie-restricted diet and increased physical activity has been recommended for individuals with obesity [14, 34, 35]. ILI with physical activity components provides various metabolic benefits, such as decreased insulin resistance, blood pressure, and improved lipid profiles, in addition to improvement in fat mass and waist circumference [34–37]. A recent posthoc analysis from the Look AHEAD trial, which was a large-scale lifestyle intervention among obese adults with type 2 diabetes [38], reported that participants who had a large increase in physical activity regardless of the group assignment had 22% (HR = 0.78, 95% CI: 0.60–1.03) lower CVD-specific mortality and a 23% (HR = 0.77, 95% CI: 0.61–0.96) lower risk of all-cause mortality, compared to those who did not increase physical activity level [17]. A similar result was observed in the Aerobics Center Longitudinal Study that within similar cardiorespiratory fitness level individuals, those with higher physical activity had a more favorable aerobic function and health profile [39]. Together with our findings, promoting physical activity regardless of body fatness can decrease CVD risk.

Biological mechanisms linking increased physical activity and lower risk of CVD are attributed to alterations in the myocardium, skeletal muscle, and vascular system [9, 40]. Physical activity is associated with increased shear stress, which leads to increased vascular nitric oxide concentration and up-regulated endothelial nitric oxide synthase activity [41, 42]. Physical activity increases the mean size of high-density lipoprotein (HDL) and low-density lipoprotein, resulting in improved endothelial function [43]. The increased shear stress can improve collateral formation angiogenesis [44, 45]. Physical activity also favorably impacts on inflammatory markers, such as decreases in C-reactive protein (CRP) and tumor necrosis factor-α (TNF-α), increasing interleukin-6 (IL6), and improved insulin sensitivity, which may contribute to cardio-protective effects [39, 46–48]. With these cardiovascular benefits and observed associations, physical activity may be treated as an independent factor for CVD risk that is not included in the Framingham Risk Score.

In this population-based sample of U.S adults, 43% of the participants that were overweight, and 53% of the participants that were obese reported being sedentary. This is consistent with prior reports of objectively-measured physical activity that the majority of overweight and obese adults do not regularly participate in the recommended levels of physical activity [49, 50]. Moreover, our findings suggest that increasing physical activity level may contribute greater risk reduction of 10-year CVD risk compared to weight status. Together with the fact that most U.S adults are classified as having a poor diet according to the Life Simple 7 dietary composite score, a substantial CVD burden could be potentially prevented by engaging in healthy behaviors. Healthcare providers play an essential role in promoting behavior change: overweight and obese adults who received advice from healthcare providers were four times more likely to attempt a healthy lifestyle compared to those who did not receive advice [51]. Although adults with diabetes, CVD, and other medical conditions receiving healthy lifestyle advice more frequently, 56–79% of overweight or obese individuals without chronic conditions reported never received lifestyle advice from their healthcare providers [52]. The observed evidence of lower CVD risk among those engaged in any physical activity among individuals who were overweight and obese suggests the possibility of engaging in a healthy lifestyle to alter the negative effects of excessive body weight on health outcomes. This highlights the gap between the urgent needs of providing healthy lifestyle advice and the current practice of healthcare providers towards overweight and obese individuals. Therefore, identifying effective strategies to deliver advice about positive health behaviors in the healthcare setting is crucial to address the needs of those populations. Perhaps training for health professionals to improve essential knowledge and skills to provide lifestyle modification advice, as well as establishing and referring to accessible community-based programs to improve physical activity level and diet quality at the population level.

It is critical to acknowledge the strengths and weaknesses of this study to facilitate the interpretation of our findings. The main strength of this study is the large sample size that is representative of the general U.S. population. It also allowed us to have enough statistical power to examine the interaction effect and joint effect of physical activity level and weight status. Additionally, the multivariable-adjusted regression models accounted for multiple variables that are known to influence the relationship between physical activity and CVD-risk, such as depression status.

The limitations of this study include the cross-sectional study design, which limited our ability to infer the causality of the observed association between physical activity and CVD risk. Because physical activity and diet quality were measured only once, it is unknown how increasing physical activity and improving diet quality would benefit cardiovascular health among overweight and obese adults. Understanding the underlying mechanism would require additional studies, perhaps through a prospective cohort study with longitudinal data to explore the temporality of the association, or a large-scale randomized lifestyle intervention trial to determine the effect of the lifestyle intervention. Also, our data were limited to a self-reported questionnaire regarding health behaviors and medical history. This likely underestimated the prevalence of comorbidities and overestimated diet quality and physical activity level. Due to the nature of the categorization, adults who reported 1–149 min/week of physical activity were all classified as physically inactive, which limited the ability to examine the variations of CVD risk within group. It is possible that adults who reported 10 min/week of physical activity had smaller CVD risk reduction than adults who reported 120 min/week of physical activity. Thus, caution is needed to interpret the results in terms of the CVD risk reduction within this group. Although aerobic exercise appears to be most beneficial at decreasing CVD risk, we were unable to distinguish aerobic or resistance exercise and the duration that would qualify as aerobic exercise from the questionnaire. The reported physical activities may include resistance exercise, which may not provide the cardiovascular benefits seen with aerobic exercise. Our analysis was limited to what was recorded in the NHANES data. It is possible that other factors, such as cardiorespiratory fitness and body composition, also play a role in CVD risk independently or interactively with physical activity and weight status [9, 53]. Future studies combining objective-measured physical activity, diet, cardiorespiratory fitness, and body composition, as well as confirmation with electronic medical records are needed to improve the quality of the data and complement the research to reduce CVD risk through health behaviors.

In conclusion, engaging in any physical activity is associated with better cardiovascular health among adults who are overweight and obese. Future studies are needed to identify the effective modes and doses of exercise that offer optimal benefit for populations by weight status.
